# Development and Validation of an Integrated HIV/STI, and Pregnancy Prevention Programme: Improving Adolescent Sexual Health Outcomes

**DOI:** 10.3390/tropicalmed10090273

**Published:** 2025-09-22

**Authors:** Mukovhe Rammela, Lufuno Makhado

**Affiliations:** 1 Department of Public Health, Faculty of Health Sciences, University of Venda, Thohoyandou, Limpopo 0950, South Africa; 2 Office of the Executive Dean, Faculty of Health Sciences, University of Venda, Thohoyandou, Limpopo 0950, South Africa; lufuno.makhado@univen.ac.za

**Keywords:** development, validation, integration, HIV, STIs, pregnancy

## Abstract

In developing countries, adolescent girls and young women (AGYW) continue to experience high rates of unintended pregnancy and sexually transmitted infections (STIs), including Human Immunodeficiency Virus (HIV). Several healthcare services are available at the primary level of healthcare to address the sexual and reproductive needs of adolescents in South Africa. Healthcare providers often face challenges such as limited resources, inadequate funds, and inadequate training, which hinder their ability to provide integrated care. Furthermore, cultural stigma and a lack of privacy prevent adolescents from seeking care. In response to increasing international calls for developing and implementing integrated person-centered care, which addresses both quality and access to care, this paper aims to develop and validate an integrated HIV/STI, and pregnancy prevention program for adolescent girls and young women in the Vhembe District of Limpopo. Multiphase mixed methods were employed in this study. This study consisted of three interconnected phases. As part of phase 1 of this study, a comprehensive literature review was conducted. In phase 2, an empirical study conducted using a concurrent triangulation strategy to collect and analyze both qualitative and quantitative data as a form of confirmation, dis-confirmation, cross-validation or corroboration of the findings. Consequently, a conceptual framework was developed using qualitative and quantitative analysis by merging, comparing, and interpreting the results. The findings of phase 2 interface were analyzed using the Political, Environmental, Social, and Technological (PEST) and Strength, Weakness, Opportunity, and Threat (SWOT) analyses. Additionally, the outcomes of the Logical Framework Analyses (LFA) informed the development of an integrated programme aimed at preventing HIV, STIs, and teenage pregnancy. Several stakeholders and experts (*n* = 35) were consulted as part of the Reduce the Risk (RTR) Coalition to validate the proposed integrated programme with an average of 94.3% on acceptability, feasibility, and appropriateness. In the Vhembe District of Limpopo province, there has been no published study that has developed an integrated HIV, STIs, and pregnancy prevention programme to improve the sexual health outcomes of adolescent girls and young women.

## 1. Introduction

Africa has the youngest population globally, with 51.7% of the South African population under the age of 25 [1]. Since the 1990s, adolescent and youth health (AYH) programs, including those on sexual and reproductive health (SRH) and youth development, have gained traction in Africa [2]. However, adolescents and youth in South Africa continue to face a high burden of morbidity and mortality from multiple factors including teenage pregnancy, unplanned pregnancy, Human Immunodeficiency Virus (HIV) and sexually transmitted infections (STIs), unsafe abortion, early and child marriage, and unmet needs for family planning [3,4,5].

Calls for effective integration of healthcare delivery systems, as a means to address equity of access and efficiency, have appeared in health reforms promoted by national governments and multilateral institutions around the world [6,7], including in South Africa. According to the World Health Organization (WHO), there were approximately 21 million pregnancies among girls aged 15 to 19 in low- and middle-income countries in 2019, with approximately 50% of these pregnancies being unintended [8]. In low- and middle-income countries, 55% of unwanted pregnancies among adolescent girls aged 15 to 19 result in abortions, which are often performed under unsafe conditions. It is estimated that 480,000 young people aged 10 to 24 were newly infected with HIV in 2022, of whom 140,000 were adolescents aged 10 to 19 [9].

Prevalence of asymptomatic curable STIs in pregnant adolescents is high and comparable with women > 20 years old. Adolescents remain at substantial risk of asymptomatic incident STIs during pregnancy [4,5]. Studies shows high prevalence and incidence of STIs in pregnancy, especially in WLHIV, demonstrating the need for STI screening to prevent adverse pregnancy and birth outcomes [10]. There is evidence that 27% of pregnancy women who are HIV-exposed have curable STIs. Pregnant women and teenagers infected with STIs had a trend towards stillbirths, possibly due to undiagnosed sexually transmitted diseases and these data highlight the importance of integrating STI testing into HIV and pregnancy prevention programs to maximize the health of women, children, and families [11].

The WHO estimates that more than one million people are infected with sexually transmitted infections (STIs) each day in the world [12]. Consequently, there are approximately 376 million cases of STIs every year, including chlamydia, syphilis, trichomoniasis, and gonorrhea [13]. Additionally, more than 500 million people live with genital infections worldwide, with herpes simplex virus (HSV) being the most prevalent [13]. In the Vhembe District, young women are disproportionately more likely to acquire these STIs than young men of the same age [14]. It has been suggested that the increased risk is caused by a combination of factors, including gender inequity in sexual relationships, early sexual debut ages, and inadequate access to sexual and reproductive health (SRH) [14].

Studies in South Africa indicate that teenage pregnancy has reached a crisis point [15]. The Vhembe District of Limpopo province has experienced an increase in teenage pregnancies, including the adolescents who are living with perinatal HIV [16]. Based on the District Health Information System [DHIS] [17] 2020 of Limpopo province, 64,372 new HIV cases have been reported in Vhembe District [17].

The Pregnant Learner Management Policy contributed to increased dropout rates as it prohibited adolescent mothers from returning to school before they had spent two years with their babies [18]. It has since been replaced with the National Policy on the Prevention and Management of Learner Pregnancy in Schools, but reintegration remains a challenge [18]. According to the literature, there has been little evidence of improvement in the coverage of HIV, STIs, teenage pregnancy prevention, and the efficiency of service delivery in South Africa [19].

Providing services through integrated and person-centered care is critical to achieving this, especially for reaching underserved and marginalized populations to ensure no one is left behind [20]. An integrated and person-centered approach has proven to be highly effective in improving delivery efficiency, reducing costs, improving equity in the uptake of services, improving health literacy and self-care, increasing patient satisfaction with care, improving patient relationships with their healthcare providers, and increasing the ability to handle healthcare crises effectively [20,21].

South Africa has made significant efforts to combat HIV and teenage pregnancy. Yet, the rates of PrEP (Pre-Exposure Prophylaxis) and contraceptive coverage among adolescents and young people are stagnating and declining [22]. The HIV prevalence rate for young people aged 15–24 is approximately 7.9% in South Africa, which is among the highest rates worldwide. Among adolescent girls and young women (AGYW) specifically, the prevalence is even higher, with around 10.9% affected. AGYW are at higher risk of HIV infection due to their lack of access to HIV prevention services, lack of education, and lack of knowledge about how to protect themselves.

Despite increasing global PrEP initiations in 2021–2024, many AGYW in South Africa who would benefit from PrEP still do not take it. As a result of a combination of factors, first, among them social factors, PrEP stigma, which discourages AGYW people from using PrEP [23]. Additionally, AGYW face challenges in effective PrEP use, which can be exacerbated by the need to keep PrEP use confidential.

Since 1998, the rate of teenage pregnancy in South Africa has remained relatively unchanged [24]. It is clear from this issue that no improvement has been made over the years. Person-centered care interventions are crucial in healthcare as they focus on the unique needs and circumstances of each individual. This approach not only improves patient satisfaction and outcomes but also fosters a more trusting and collaborative relationship between healthcare providers and patients [25]. In the context of teenage pregnancy and HIV/STI, person-centered care can lead to more effective and tailored interventions that address the specific challenges faced by youth in Vhembe District. Additionally, interventions should consider social, economic, and cultural factors that impact adolescents and young people’s access to services.

Based on the literature, no person-centered care interventions have been made available to address teenage pregnancy and HIV/STI among youth in Vhembe District. Risk factors for STIs including HIV may differ between age groups of girls and women, and mitigation interventions may need to be tailored accordingly [26]. Several structural and social factors are preventing the implementation of the existing programs in Vhembe District from meeting the Departmental set targets [27]. Thus, this paper aims to develop and validate an integrated HIV/STI and pregnancy prevention programme.

## 2. Materials and Methods

**Figure 1:**
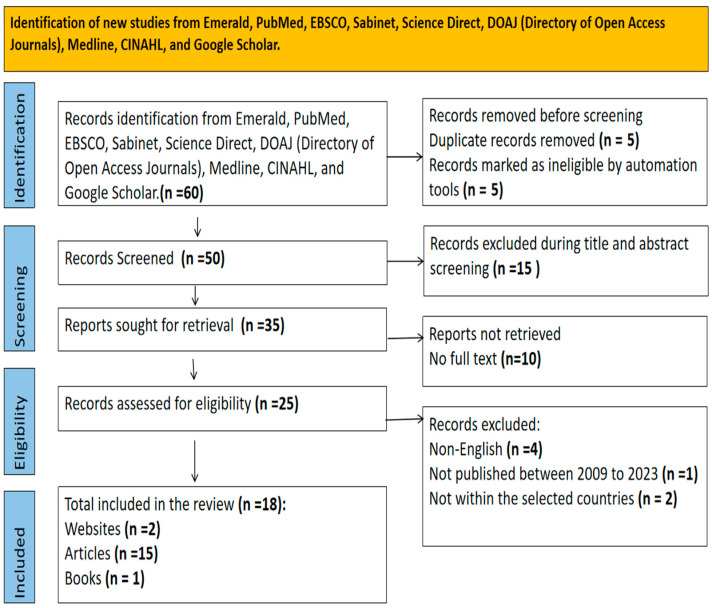
PRISMA Flow Chart.

A multiphase mixed-method approach was employed to achieve the objective of this research. The development of the programme was conducted in separate phases. A preliminary analysis of both comprehensive literature review on available strategies to integrate HIV, STI and pregnancy prevention (shown in Figure 1) and empirical studies was conducted in the first phase. The preliminary analysis of both comprehensive literature review, qualitative and quantitative studies involved a structured approach to data collection and interpretation. The exploratory qualitative data were presented in terms of adolescents’ and healthcare providers interview responses, which were classified into different themes. The quantitative cross-sectional data were presented in descriptive statistical analysis. The results were interpreted and merged, providing a comprehensive understanding of the research outcomes (shown in Table 1).

The PEST–SWOT analysis for the second phase was established. Phase two was built on the merged data analysis from phase one to inform programme development. It was guided by available resources to ensure that the programme meets the needs of the adolescents in the Vhembe District. The integrated programme was developed to accommodate adolescents’ changing needs.

The review in Figure 1, examines the effectiveness of promoting reproductive health outcomes in young women by providing integrated HIV/STI prevention into family planning and contraception services under one programmatic umbrella. The comprehensive literature review highlights the widespread acceptance of integrating HIV/STI, into pregnancy prevention services, even in resource-limited healthcare systems. The review identified several approaches to providing integrated services, including comprehensive care packages, referrals, and models that effectively link services. The review also emphasizes the need to carefully plan and implement integrated services based on current scientific literature and technical guidance.

### 2.1. Preliminary Analysis

#### 2.1.1. Merging Data from Four Papers

To provide a comprehensive analysis of how teenage pregnancy, sexually transmitted infections, and HIV infection among youth are addressed in the Vhembe District, the findings from these four papers were compared side by side. Thus, a merged output was generated so that an overview of key components that need to be considered and addressed in the development of the program could be obtained. The first paper conducted a comprehensive literature review to examine HIV, STI, and teenage pregnancy prevention (family planning) integration strategies nationally and internationally. In the second paper, we evaluated the implementation of adolescent and youth-friendly services (AYFS) in selected primary healthcare facilities in Vhembe District, Limpopo. The third paper explored teenagers’ experiences and challenges of accessing HIV PrEP services among family planning users at selected facilities in the Vhembe District. In the final study, barriers and facilitators of integrating HIV PrEP into family planning services for improving sexual health outcomes of young people at selected health facilities in Vhembe District.

#### 2.1.2. PEST Analysis

PEST (Political, Economic, Socio-Cultural, and Technological) analyses were conducted to suggest and evaluate integrated prevention program ideas. The analysis of PEST factors helped to identify potential interventions [28]. It also provided insights into how the program will be implemented and monitored. Additionally, it also provided guidance on how the program could be adapted to changing external conditions. During the development of a programme, it is imperative to ensure that the plan aligns with the Department of Health’s mission and objectives rather than working against them.

#### 2.1.3. SWOT Analysis

Merging the empirical findings was conducted using the SWOT analysis method. Based on the findings of three papers, this method provided insight into the strengths and weaknesses of the current implemented programmes in preventing HIV and pregnancy among youth in Vhembe District. The SWOT analysis also provided recommendations on how to introduce comprehensive, integrated measures to prevent HIV and teenage pregnancy. The recommendations were incorporated to develop an integrated HIV and pregnancy prevention programme for Vhembe District.

**Figure 2:**
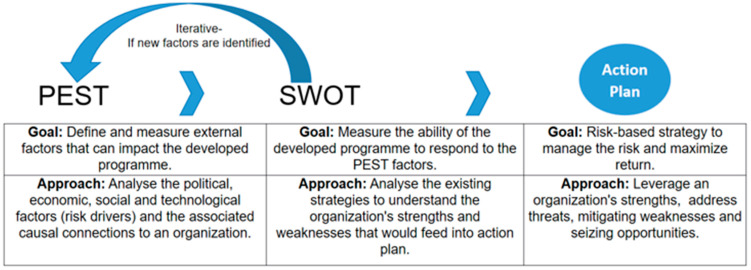
Overview of Integrated PEST–SWOT.

Integrating PEST and SWOT analysis provided a holistic strategic perspective, as shown in Figure 2. In addition to identifying the external factors that may affect operations, this approach also allowed the assessment of internal strengths and weaknesses. Consequently, this dual analysis helped in making more effective strategic decisions and ensuring that the programme would be feasible and successful. SWOT analysis also helped to identify opportunities for successful implementation of the programme [29].

## 3. Results

This step compared the findings from the four papers side by side to have a comprehensive overview of the state of matters concerning adolescent sexual practices. By intentionally integrating data in Table 1, the researcher aims to gain the knowledge or insights unavailable to a quantitative or qualitative study undertaken independently.

**Table 1:** Merged Findings.

First Paper Findings (Comprehensive Literature Review)	Second Paper Finding (Quantitative Cross-Sectional Study)	Third Paper Findings (Exploratory Qualitative Study)	Fourth Paper Findings (Exploratory Qualitative Study)	Merged Analysis Findings
Contributing factors ✓ Acceptance of integrating HIV, STI, and pregnancy prevention services, even in resource-limited healthcare systems [30,31,32,33]. ✓ Effective referral models [34]. ✓ Capacity building for healthcare workers [35,36]. ✓ Providing adequate commodities [37]. ✓ Integration of community outreach interventions [38]. ✓ Lack of integrated national policies [39]. ✓ Health service budgets and supplies are limited [37,40,41]. ✓ Limited trained staff [42]. ✓ Negative attitude of HCPs towards service users [43]. ✓ Lack of knowledge regarding PrEP and family planning [44]. ✓ Collaboration with NGOs [45].	✓ Standard 1: Provision of adolescent health literacy. The use of IEC materials found to be relatively low at 36% (*n* = 112) and the implementation of AYFS signage and branding with a score below 31% (*n* = 112) [27]. ✓ Standard 2: Community support engagement levels were found to be relatively low, with only 21.5% (*n* = 112) of healthcare providers engaging with the parents or guardians of young clients. ✓ Standard 3: Appropriate package of services—Awareness of the AYFS service package among healthcare providers is approximately 48.2% (*n* = 112). ✓ Standard 4: Provider competency—Significantly fewer have received specific training in AYFS 16% (*n* = 112) or on Pre-Exposure Prophylaxis (PrEP) 25.9% (*n* = 112). ✓ Standard 5: Facility characteristics—Facilities demonstrate 100% commitment to accessibility by operating over weekends, and insufficient allocation 46.4% (*n* = 112) of specific time slots for adolescents. ✓ Standard 6: Equity and non-discrimination—80% (*n* = 112) adherence to privacy guidelines protecting adolescent confidentiality. ✓ Standard 7: Data and quality improvement—Healthcare providers 51.8% (*n* = 112) receive training in monitoring AYFS performance. ✓ Standard 8: Adolescent participation—The participation and involvement of adolescents in the planning of their services is low 25.9% (*n* = 112).	Institutional challenges encountered. ✓ Medicine Stock-Out. ✓ Shortage of qualified nurses for PrEP distribution and administration. ✓ Long waiting times at the clinics. ✓ Negative attitudes among nurses toward adolescent sexuality and PrEP use. ✓ Parental consent as a requirement for receiving PrEP. Social challenges encountered. ✓ Fear of inadvertent disclosure to family and partners. ✓ Community stigma on PrEP use. Strategies to foster the integration of PrEP programme into family planning. ✓ Availability PrEP and FP trained HCPs. ✓ Sensitizing the community about PrEP. ✓ Collaborative effort between stakeholders.	Barriers to integrate HIV prevention into family planning services. ✓ Ineffective implementation and monitoring of existing services for adolescents in clinics. ✓ Limited knowledge among healthcare workers. ✓ Shortage of staff and staff absenteeism. ✓ Shortage of essential supplies. ✓ Lack of funding for certain aspects of the services. ✓ Lack of training in PrEP. Facilitators to integrate HIV prevention into family planning services. ✓ Community support. ✓ Motivated staff. ✓ Technical support. ✓ Stakeholder collaboration. Overcoming the barriers. ✓ Improvement of monitoring the existing services for adolescents in clinics.	Key factors driving PrEP and FP services. ✓ There is a need to consider factors, causes, and potential outcomes when developing an integrated programme. ✓ AYP-related issues are handled in different contexts by different stakeholders. The nature of programmes implemented by different healthcare providers. ✓ The district suffers from structural and systemic factors that hinder AYFS provision and implementation. ✓ As a result of the current setup, PrEP and FP services run in parallel. The impact of challenges faced by teenagers when accessing PrEP services. ✓ Poor service delivery. ✓ HCP’s negative attitude. ✓ Shortages of medication. Possibility of developing an integrated programme. ✓ From the findings obtained, there is a high probability that the services can be integrated as the different stakeholders realize the benefits of collaboration. ✓ Challenges that could hinder this integration and ways of overcoming them were suggested. ✓ Limited PrEP knowledge, lack of resources and parallel services pose a threat to integration. ✓ Continuous improvement focusing on improving AYFS quality for adolescents. ✓ Develop an integrated program using a person-centered approach. ✓ Developing a program that takes local circumstances into account. ✓ Service providers take responsibility for providing quality services to adolescents and young people.

### 3.1. PEST Analysis Outcome

To design an effective program that ensures a positive impact on the lives of impacted population, it is crucial to address in detail the critical factors identified through the PEST–SWOT analysis. This exhaustive analysis in Table 2 reveals a series of threats, opportunities, weaknesses and strengths that shape the prevention in the context of HIV, STI and teenage pregnancy. In the political sphere, threats arising from poor infrastructure and Non-compliance to national guidelines and SOPs suggest the constant need for adaptation and regulatory compliance by healthcare institutions. This not only involves operational adjustments, but also strategic investments to ensure the safety and privacy of patients information in a digitally changing environment. Likewise, political instability can affect the long-term planning of integrated prevention program, complicating investment in infrastructure and the effective implementation of integrated innovations. From an economic perspective, limited budget for staff training stand out as significant challenges. Socially, community resistance and stigma of PrEP and healthcare provider’s attitude towards service users are key obstacles. In the technological field, evidence is presented that poor compliance with guidelines and SOPs impacts the effectiveness of health services provision and leads to teenagers’ unmet health needs. The findings revealed that these factors are interconnected and that all of them must be addressed in parallel to effectively reduce the prevalence of teenage pregnancy and HIV/STI among the youth. Additionally, the results showed that extrinsic factors including community resistance and stigma and internal factors such as healthcare provider’s attitude towards service users and limited budget for staff training are the most important factors in HIV, STI and pregnancy prevention service integration, respectively, so it is recommended that the most attention and efforts for service provision be focused on improving these factors and especially internal factors. 

### 3.2. SWOT Analysis Outcome

In this study, the results were analyzed in detail to identify the strengths, weaknesses, opportunities, and threats associated with the prevalence of teenage pregnancy and HIV/STI among youth in the Vhembe District as shown in Table 3. The empirical findings provide evidence on challenges unique to low-resource, rural settings, contributing to global discourse on HIV and SRH integration as shown in Table 3. Following the analysis, it calls for the integration of PrEP, PEP into family planning routine services and highlights the need for national guidelines on minor consent for PrEP in the local clinics. It underscores the importance of a multi-sectoral, integrated prevention program linking schools, parents, NGOs, social clubs, and healthcare providers. Consequently, it embeds structural improvements on nurse-patient ratios, real-time stock control, updated training and robust monitoring systems.

### 3.3. Application of the Logical Framework Analysis (LFA)

**Figure 3:**
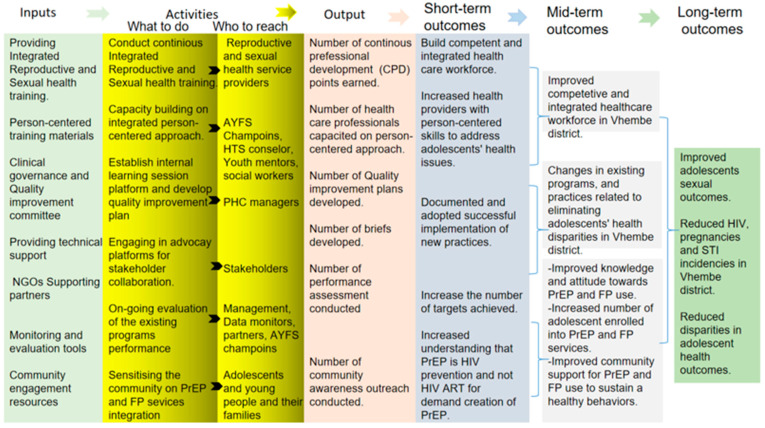
Application of the Logical Framework Analysis (LFA).

Therefore, Logical Framework Analyses (LFAs) are extensively utilized by governments and non-governmental organizations in the planning and development of programs. The factors related to fragmented service delivery were evaluated by pairwise comparisons during the PEST–SWOT analysis and based on them, the planning and monitoring matrix were created. This matrix included general and objective goals, as well as control activities and indicators. Based on the LFA methodology (Figure 3), the key components of the program were analyzed in order to identify the problems and as a result, prepare the Mitigation plans. The results of the LFA shown in Figure 3, provide a model to planning and designing for integrated preventive program.

### 3.4. Programme Design

At this stage, the SWOT (Table 3) and PEST analysis findings (Table 2) were used and LFA outcomes in Figure 3, which include the need for integrated reproductive and sexual health training (AYFS, PrEP, PEP, PICT, SRHR and CTOP), community and healthcare workers capacity building on an integrated Ubuntu approach, establishment of an internal learning session platform and developing quality improvement plans, engagement in advocacy platforms and strengthening stakeholder collaboration, on-going assessment of the existing programs performance and sensitizing the community about PrEP, condoms and modern contraceptives services to design the programme content. During the investigation, the researchers were guided by Santana’s model [46], Milio’s prevention model [47], and Donabedian’s SPO model [48] which helps to identify areas where adolescent health services can be integrated and improved, such as patient experience, system performance, and service delivery. Therefore, the programme design adopted a person-centered approach to integrating HIV, STI and pregnancy prevention services for improving adolescents’ sexual health outcomes (see Table 4).

### 3.5. Programme Validation

#### Reducing the Risk (RTR) Coalition Outcome

A valid programme can achieve desired goals by determining feasibility, applicability, acceptability, and sustainability [49]. At this stage, service users and providers were consulted for listening sessions on the proposed programme as part of the Reducing the Risk (RTR) Coalition. A listening session was held with community stakeholders, nurses, community health workers, local teens, parents, and schoolteachers.

During the RTR Coalition, all participants were presented with the findings from the empirical study in Table 1, the SWOT in Table 3, PEST analysis in Table 2, and the proposed programme in Table 4. Using this method, participants’ opinions were obtained on the proposed programme (see Table 5) feasibility, applicability, acceptability, and sustainability to determine whether they will affect achieving the set goals and objectives [50]. The feedback from the participants was analyzed to customize the programme.

To ensure that all targeted categories of key stakeholders were represented, 35 stakeholders were sampled using purposive sampling, see Table 5. By using a checklist with 07 questions in Table 6, key stakeholders and experts were able to provide their input regarding the proposed programme’s appropriateness, feasibility, accessibility, and sustainability. A response rate of 100% was achieved and the data saturation level was reached after the last 10 stakeholders provided the same answers. Their responses were analyzed and then used to customize the approved programme development in Table 7. In the course of the validation sessions, it was observed that most stakeholders expressed a desire to work collaboratively to ensure the success of the integrated programme. They emphasized the importance of open communication and mutual support to address challenges and achieve shared goals. This collaborative approach was seen as essential for ensuring the program’s long-term sustainability and effectiveness across all the involved clinics.

A revised integrated HIV, STI and pregnancy prevention programme as shown in Table 7, will be implemented with improved reporting systems between clinics, schools and SAPS for pregnant teenagers infected with HIV under 16 years of age for further investigation. It also seeks to strengthen coordination between healthcare providers and law enforcement to better protect and support at-risk youth. The program will also focus on outreach efforts aimed at reducing stigma associated with PrEP, PEP and contraceptives, and promoting healthy behaviors among adolescents.

The stakeholders’ responses were carefully analyzed and then utilized to improve and refine the approved programme in Table 7. This allowed for a more inclusive and effective programme that addressed the needs and concerns of all stakeholders involved.

Engaging stakeholders in the validation process ensures that their diverse perspectives and expertise are considered, leading to more sustainable and well-rounded programme. Over time, this collaboration fosters trust and strengthens relationships, which can enhance community support and programme success. Furthermore, ongoing stakeholder involvement encourages adaptability and innovation, helping the programme remain relevant and effective in a changing environment.

## 4. Discussion

The empirical findings provide evidence on challenges unique to low-resource, rural settings, contributing to global discourse on HIV and SRH integration as shown in Table 1**.** Several AYFS intervention strategies have not been successfully implemented in the District [27]. In consequence, adolescents are at greater risk of HIV and STI transmission, and pregnancy as a result of a lack of access to integrated services. According to the District Health Information System [DHIS] [17] 2020, 64,372 new HIV cases have been reported in the Vhembe District of Limpopo province, representing approximately 11.4% of the total population. The Limpopo Department of Health reports that, in each of the two secondary schools outside Thohoyandou, there are 36 pregnant learners, and five of these students are HIV positive [51]. A report indicates that 31 additional students, ages nine to 19, from both primary and secondary schools in the same area have been infected with HIV. Consequently, the department has had difficulty preventing the spread of HIV among the youth in the Vhembe District.

In Vhembe District, access to sexual and reproductive healthcare is hindered by several barriers, according to Mulaudzi [52]. In health facilities, HIV PrEP and contraceptives are not widely known to patients, and this has affected their utilization of these services. Furthermore, adolescents have been discouraged from using contraceptives due to inadequate facilities and equipment, disruptions in supplies, a lack of information provided to clients, and providers’ insensitivity to their feelings and needs [52]. It has been recognized that inadequate infrastructure, including multiple and poorly equipped consultation rooms, negative attitudes among health workers, poor communication, high patient loads, inadequate staffing, and a lack of knowledge among service providers, particularly concerning long-term family planning, pose significant challenges to the integration of services. Furthermore, essential supplies such as HIV test kits, drugs, and gloves are in short supply [53].

As a result of the merged analysis in, HIV/STI prevention and family planning programs targeting adolescents run parallel, although they are complementary in some ways. Integration of sexual health services into primary healthcare is essential to improving young people’s utilization of sexual health services and to improving HIV testing interventions [38]. The integration of PrEP with other sexual and reproductive health (SRH) services can have a significant impact on the health of young women. Both providers and clients may benefit from the integration of PrEP, PEP into family planning routine services. Furthermore, the merged analysis indicates that it is feasible to integrate HIV, STI, and pregnancy prevention services, as demonstrated by the motivation and support of different stakeholders.

The SWOT analysis in Table 3 indicates (as a strength) that stakeholders are committed to collaboration. It was apparent that all stakeholders were committed to working together and collaborating regardless of their differences in background. Potential threats include lacking resources, stakeholder knowledge, and poor compliance with government guidelines and policies. Applying the logical framework approach highlighted key steps and interventions that must be considered in developing and implementing the programme. There is a need to ensure that there is sufficient funding and human resources to conduct training and community outreach, provide referrals between the community, school and linked clinic for teenagers at risk of HIV, STIs, and pregnancy, and improve communication between providers and patients.

Additionally, communities should be ensured that they are involved and engaged in integration activities, focusing on at-risk groups such as adolescents through school-clinic referrals [54]. Establishing clear communication channels between schools and clinics is important, as well as ensuring that both parties know the referral processes and criteria. Training school staff on identifying at-risk adolescents and understanding the referral system can enhance effectiveness [55,56]. Additionally, involving parents and guardians in the process can foster a supportive environment for the adolescents.

As a result of sexual abuse, HIV and STI incidences as well as unwanted pregnancies and illegal abortions are on the rise worldwide [57]. Recent literature suggests that all perpetrators of sexual abuse can be criminalized and prosecuted [57]. Kavanagh et al. [58] in their paper, identified law reform as a critical pandemic intervention since global goals to decrease HIV incidence and mortality were not met. Consequently, this study proposed that healthcare providers, social workers, teachers and parents of teenagers should report all the cases of any teenager impregnated or infected with HIV by an adult to the SAPS as statutory rape for further investigation by using Form 22 (see Supplementary Materials) that complies with POPIA/GDPR standards. Breckenridge et al. [59] also highlights workforce development and coordination across health service systems to provide holistic support for victims and survivors. Morrissey [60] emphasized that information and education about the legal system and legal rights should be promoted; and protection for survivors must be enhanced.

The integration process must be conducted in such a way that the intended outcomes of such integration are made clear and understood by all the stakeholders involved in the program [61]. As a result, the developed programme aims to improve adolescents’ access to integrated HIV, STI, and pregnancy prevention services at the primary point of care. Sub-Saharan Africa (SSA) has reported several barriers to accessing healthcare, including lack of resources, knowledge gaps, training gaps, and parallel health systems [62]. In addition to improving health outcomes, adolescents will be able to access integrated HIV, STI, and pregnancy prevention health services through the development of this program in Table 7, the study findings underscores the importance of a multi-sectoral, integrated prevention program linking schools, parents, NGOs, social clubs, and healthcare providers. Consequently, it embeds structural improvements on nurse-patient ratios, real-time stock control, updated training and robust monitoring systems. This research provided evidence that the negative attitudes of nurses and teachers as a barrier for teenagers to access sexual reproductive health (SRH) and contraceptive services, congruent to other studies [63,64]. Teachers, nurses, and community members need to collaborate to address learners’ reproductive health needs [54]. Thus, there is a need to incorporate Ubuntu philosophy in the prevention of HIV/STI and teenage pregnancy to facilitate collaborative and inclusive actions to address the prevention of teenage pregnancy in Vhembe District. It is common practice in Ubuntu to invest in collective actions in order to better one’s life, which reinforces the need for shared identity and solidarity among its members [65]. An integrated service approach has proven to be highly effective in improving delivery efficiency, improving equity in the uptake of services, enhancing health literacy and increasing patient satisfaction with care, strengthening patient relationships with their healthcare providers, and increasing the ability to handle healthcare crises effectively [21].

Table 7, the integrated service approaches can reduce health disparities and improve the quality of care [21]. Therefore, this study highlights the importance of integrating STI testing into HIV and pregnancy prevention programs to maximize the health of women, adolescents, and families. Integrated service approaches recognize the interconnectedness of sexual and reproductive health, social determinants of health, and other factors contributing to health outcomes. Evidence shows that previously developed integrated HIV, STI, and pregnancy prevention interventions improve population health outcomes [38,66]. The integrated HIV, STI and pregnancy prevention programme provides more comprehensive and holistic care tailored to the individual’s needs. Healthcare providers play a crucial role in the success of integrated service approaches by coordinating and collaborating across different disciplines to deliver comprehensive care. Furthermore, healthcare providers are key to building trust and maintaining open communication with patients, which is essential for understanding their unique circumstances and effectively tailoring care plans. It is evident that these interventions can be effective in preventing HIV, STIs, and unplanned pregnancies all at the same time. Tailored approaches that address cultural and social factors are crucial for maximizing their impact and ensuring long-term success in promoting public health.

## 5. Limitations

A comprehensive literature review was conducted using secondary data collected in different contexts in the Sub-Saharan Africa countries. During data collection in this study, there was no randomization involved, as this would negate the study purpose. Researchers would not benefit from speaking with healthcare providers who do not offer sexual and reproductive healthcare services when the information needed for this study is specific to their area of expertise.

## 6. Conclusions

In conclusion, the findings of this study indicate that AYFS implementation is limited in the Vhembe District due to training deficiencies, staff attitude and community stigma which are the leading causes of poor access to HIV, STI and pregnancy prevention services. This highlights the urgent need for the integration of HIV, STIs, and pregnancy prevention programme to address these gaps and improve service delivery, given that all stakeholders demonstrated a readiness to participate in such a programme. The stakeholders agreed that such a programme would be an effective way of reducing HIV, STI transmission and teenage pregnancies while also providing reproductive health services. The stakeholders have expressed a desire to work collaboratively to ensure the success of the integrated programme. To implement the integrated programme, stakeholders could establish joint training sessions for healthcare providers to ensure consistent service delivery. They could also create a centralized platform for sharing resources and best practices. Additionally, regular community and school outreach events could be organized to raise awareness and encourage participation in the programme. Integrating HIV/STI and pregnancy prevention can contribute to a sustainable response to the HIV epidemic, streamline service delivery, and improve the health outcomes and lives of AGYW.

## Figures and Tables

**Table 2:** PEST Analysis Findings.

Political Factors ✓ Poor infrastructure.	Economic Factors ✓ Budget constraints for essentials and services such as lab costs of blood services and community campaigns. ✓ Limited budget for staff training and hiring.	Implications for programme goals ✓ Negative impact on operational service delivery. ✓ Patients appointments postponed or cancelled.
Social Factors ✓ Overcrowded facilities. ✓ Community resistance and stigma of PrEP. ✓ Healthcare provider’s attitude towards service users.	Technological Factors ✓ Non-compliance to national guidelines and SOPs. ✓ Poor utilization of the Department of Health e-learning through Knowledge Hub.	Implications for programme goals ✓ Reduction in patients’ treatment satisfaction. ✓ Negative impact of patient health outcomes.

**Table 3:** SWOT Analysis Findings.

Strengths ✓ AYFS and PrEP services are recognized in Vhembe District ✓ Stakeholder collaborations	Weakness ✓ PrEP and FP run parallel in the facilities. ✓ Non-adherence to recognized AYFS guidelines. ✓ Limited implementation of the AYFS programme in Vhembe District. ✓ Ineffective monitoring of existing services for adolescents in the clinics. ✓ Delayed implementation of PrEP in Vhembe District.
Opportunities ✓ Community support ✓ Staff motivated to integrate PrEP and FP if trained	Threats ✓ Shortage of staff and absenteeism. ✓ Limited knowledge on AYFS, PrEP and other FP services. ✓ Healthcare providers’ negative attitude. ✓ Lack of funding for certain aspects of adolescent health services.

**Table 4:** Key Components of Integrated HIV/STI and Pregnancy (IHSP) Prevention Programme.

Proposed Interventions	Programme Goals	Planned Activities	Indicator Targets	Responsible Stakeholders	Expected Outcomes
Incorporation of Ubuntu principles and values into HIV, STI and pregnancy prevention service.	Implementing HIV, STI and pregnancy prevention services that address adolescents’ health disparities and improve their sexual health outcomes through mutual care and interconnectedness with the providers.	Strengthening community involvement and empathetic communication for healthcare providers can create trust and encourage young people to seek necessary care without fear of judgment.	10% of Ubuntu trained nurses per facility.	Ubuntu trained HIV, STIs, and pregnancy prevention healthcare providers.	Reduced stigma associated with HIV PrEP, Condom and Contraceptive use. Improved health outcomes for adolescents.
Competent integrated HIV, STI and pregnancy prevention workforce.	Ensure universal service delivery and access to quality sexual and reproductive healthcare services.	Provide integrated HIV, STI and pregnancy prevention training for healthcare providers in remote and underserved areas. Increasing workforce knowledge and skills related to HIV, STIs, and pregnancy prevention through e-learning (Department of Health Knowledge Hub platform).	10% of AYFS-certified nurses per facility 10% of PrEP-certified nurses per facility 10% of PEP-certified nurses per facility.	District sexual and reproductive health coordinators.	Improved quality of HIV, STI and pregnancy prevention services.
Integrated HIV, STI and pregnancy prevention community-based training and engagement.	Assisting community members and adolescents, retaining knowledge, acquiring skills, and preparing for a quality future life.	Providing comprehensive (HIV, STI and pregnancy prevention) education and outreach programs and partnerships with local organizations to empower adolescents to make informed decisions about their sexual and reproductive health. Expanding mobile health clinics to reach remote areas and underserved communities.	4 Community-outreach campaigns conducted per month.	Health Service Providers and Vhembe District supporting partners (NGOs).	Reduced prevalence of cases of HIV, STIs and teenage pregnancy due to a sexually healthy lifestyle.
Integrated HIV, STI and pregnancy prevention community-school referral systems.	To bridge the gap between community, local schools and health services, allowing for better communication and coordination.	Support and train schoolteachers and community stakeholders to identify adolescents at risk of HIV, STI and pregnancy and refer them to the linked facility.	4 School-outreach campaigns conducted per month.	School teachers, community stakeholders and healthcare providers.	Reduced prevalence of cases of HIV, STIs and teenage pregnancy at Vhembe District schools.
Establishment of integrated HIV, STI and pregnancy prevention clinical governance committee.	Ensure staff compliance with global standards and policies relating to HIV, STI and pregnancy prevention services.	Developing strategies to improve access to and quality HIV, STI and pregnancy prevention services.	4 Quality improvement plans on dual-method uptake.	District sexual and reproductive health coordinators.	Improved access to quality HIV, STI and pregnancy prevention services.
Enhanced monitoring and evaluation of integrated HIV, STI and pregnancy prevention services.	Achieve the HIV, STI and pregnancy prevention indicator targets.	Measure the impact of the interventions, identify improvement areas, and ensure sustainability accountability.	90% PrEP/PEP initiation rate per adolescent visits headcount.	Monitoring and evaluation of trained health providers.	Improved quality of services offered.

**Table 5:** Stakeholders and Experts Demographics.

Variables	Frequencies *N* = 35	Percentages %
Gender		
Males Females	26 9	74.3 25.7
Participant Group		
Healthcare Providers Community Leaders School Principals Parent/Guardian Adolescents Researchers	12 3 3 7 6 4	34.3 8.6 8.6 20 17.1 11.4
Age Group		
15–19 20–29 30–39 40–49 50–59 60-above	6 4 16 5 3 1	17.1 11.4 45.7 14.3 8.6 2.9

**Table 6:** Key Stakeholders and Experts Checklist Questionnaire of the Proposed Programme (Adapted from: Proctor et al.) [50].

Statements	Response (*N* = 35)		
	Yes (%)	No (%)	Comments
Feasibility: Is the proposed programme suitable? Is there practicality toward implementation?	33 (94.3)	2 (5.7)	A lack of transportation in the Department of Health is preventing healthcare providers from reaching the intended population through community outreach and school health programs. Once this issue has been resolved, all will be in place to reach the intended population.
Accessibility: Is the programme agreeable amongst the stakeholders?	33 (94.3)	2 (5.7)	To ensure human rights protection for adolescents, SAPS services should be integrated into HIV, STI, and teenage pregnancy prevention programs.
Appropriateness: Considering the setting and target audience, is the programme relevant?	33 (94.3)	2 (5.7)	Due to the lack of accountability for those infecting and impregnating teenagers, the root cause is not addressed holistically. Therefore, NDoH should collaborate with SAPS to conduct a thorough investigation and ensure justice.
Adoption: Is there an intention to adopt the developed programme?	32 (91.4)	3 (8.6)	MEC of Health should regularly conduct unannounced visits to rural clinics where there is high teenage pregnancy and new HIV infections to ensure the quality of healthcare services are provided. This will help to identify any area that requires improvement, and healthcare providers will ensure accountability for their actions.
Coverage: Is the desired population eligible to receive and benefit from the programme?	34 (97.1)	1 (2.9)	The SAPS should be notified about all teenagers under 16 years who have been impregnated and infected with HIV by any person above 16 years old. A parent should open the case, and a teenager should be referred by a nurse or social worker for further investigation.
Fidelity: Will the intervention be delivered as intended?	29(82.9)	6 (17.1)	The challenge will be the severe shortage of staff where passionate nurses will find it hard to implement the proposed interventions due to the abnormal staff ratio at our clinics.
Sustainability: Are the programme interventions sustainable?	31 (88.6)	4 (11.4)	The majority of nurses in our clinic are older, which makes it difficult to implement the proposed framework for improving the nurse-patient relationship; we need more nurses who are younger, friendly and approachable by youth.

**Table 7:** Refined Integrated HIV, STI and Pregnancy Prevention Programme for Implementation.

Proposed Interventions	Programme Goals	Planned Activities	Indicator Targets	Responsible Stakeholders	Expected Outcomes
Incorporation of Ubuntu principles and values into HIV, STI and pregnancy prevention service.	Implementing HIV, STI and pregnancy prevention services that address adolescents’ health disparities and improve their sexual health outcomes through mutual care and interconnectedness with the providers.	Strengthening community involvement and empathetic communication for healthcare providers can create trust and encourage young people to seek necessary care without fear of judgment.	10% of Ubuntu trained nurses per facility.	Ubuntu trained HIV, STIs, and pregnancy prevention healthcare providers.	Reduced stigma associated with HIV PrEP, Condom and Contraceptive use. Improved sexual and reproductive health outcomes for adolescents.
Competent integrated HIV, STI and pregnancy prevention workforce.	Ensure universal service delivery and access to quality sexual and reproductive healthcare services.	Provide integrated HIV, STI and pregnancy prevention training for healthcare providers in remote and underserved areas. Increasing workforce knowledge and skills related to HIV, STIs, and pregnancy prevention through e-learning (Department of Health Knowledge Hub platform).	10% of AYFS-certified nurses per facility 10% of PrEP-certified nurses per facility 10% of PEP-certified nurses per facility.	District sexual and reproductive health coordinators.	Improved quality of HIV, STI and pregnancy prevention services across all the facilities.
Integrated HIV, STI and pregnancy prevention community-based training and engagement.	Assisting community members and adolescents, retaining knowledge, acquiring skills, and preparing for a quality future life.	Adolescents can be empowered to make informed decisions about their sexual and reproductive health through comprehensive education and outreach programs, as well as the establishment of partnerships with local organizations. Furthermore, expanding mobile health clinics can help reach remote areas and underserved populations.	4 Community-outreach campaigns conducted per month.	Health Service Providers and Vhembe District supporting partners (NGOs).	Reduced prevalence of cases of HIV, STIs and teenage pregnancy due to a sexually healthy lifestyle.
Integrated HIV, STI and pregnancy prevention community-school referral systems.	To bridge the gap between community, local schools and health services, allowing for better communication and coordination.	Support and train schoolteachers and community stakeholders to identify adolescents at risk of HIV, STI and pregnancy and refer them to the linked facility.	4 School-outreach campaigns conducted per month.	School teachers, community stakeholders and healthcare providers.	Reduced prevalence of cases of HIV, STIs and teenage pregnancy at Vhembe District schools.
Establishment of integrated HIV, STI and pregnancy prevention clinical governance committee.	Ensure staff compliance with global standards and policies relating to HIV, STI and pregnancy prevention services.	Developing strategies to improve access to and quality HIV, STI and pregnancy prevention services.	4 Quality improvement plans on dual-method uptake.	District sexual and reproductive health coordinators.	Improved access to quality HIV, STI and pregnancy prevention services.
Enhanced monitoring and evaluation of integrated HIV, STI and pregnancy prevention services.	Achieve the HIV, STI and pregnancy prevention indicator targets.	Measure the impact of the interventions, identify improvement areas, and ensure sustainability accountability.	90% PrEP/PEP initiation rate per adolescent visits headcount.	Monitoring and evaluation of trained health providers.	Improved quality of services offered.
Establishment of a Risk Committee in each community to address teenage pregnancy and HIV.	To identify, assess, and manage the risks of HIV and pregnancy among adolescents and build healthier and safer environments for teens.	The Risk Committee assesses the community’s risks and develops strategies to mitigate those risks. They should also create an emergency plan and ensure all stakeholders are aware of it and prepared to act if needed.	90% adolescents referred to the clinic for HIV/STI screening and PrEP/PEP initiation per referred total headcount.	Parents, teachers, healthcare providers, community leaders, local businesses, law enforcement, and other stakeholders.	Safe environment/community that reduces HIV, STIs, and teenage pregnancy.
Enhanced reporting system between clinics and SAPS for children under the age of 16 infected with HIV or impregnated by an adult using form 22 (see Supplementary File).	Ensure that teenagers are protected, and they receive the care and support they need.	District program coordinators to provide support and training to healthcare providers, social workers, teachers and parents of teenagers regarding the process flow for reporting statutory rape for further investigation of any teenager impregnated or infected with HIV by an adult.	90% of all adolescents under the age of 16 infected with HIV or impregnated by adults referred to the nearest SAPS for further investigation.	Parents, teachers, healthcare providers, community leaders, law enforcement, and other stakeholders.	Reduced prevalence of cases of HIV, STIs and teenage pregnancy at Vhembe District local communities.

## Data Availability

Data presented in this study can be obtained from the corresponding author upon request. Due to this study’s sensitivity and ethical clearance conditions, the data are not publicly available.

## Supplementary Materials

The following supporting information can be downloaded at: https://www.mdpi.com/article/10.3390/tropicalmed10090273/s1, File S1: Form 22 reporting of abuse or deliberate neglect of child.
