# Supplementary material for: Development and Validation of an Integrated HIV/STI, and Pregnancy Prevention Programme: Improving Adolescent Sexual Health Outcomes
Source: Trop Med Infect Dis. 2025 Sep 22;10(9):273. doi: 10.3390/tropicalmed10090273 (PMC12474225; doi:10.3390/tropicalmed10090273)
Supplement: Supplementary file 1 [file tropicalmed-10-00273-s001.zip › tropicalmed-3781262-SI.pdf]

REPORTING OF ABUSE OR DELIBERATE NEGLECT OF CHILD
(Regulation 33)
[SECTION 110 OF THE CHILDREN'S ACT 38 OF 2005]

REPORTING OF ABUSE TO PROVINCIAL DEPARTMENT OF SOCIAL DEVELOPMENT, DESIGNATED CHILD PROTECTION ORGANISATION OR POLICE OFFICIAL

NOTE: A SEPARATE FORM MUST BE COMPLETED FOR EACH CHILD

TO: The Head of the Department

Pursuant to section 110 of the Children's Act, 2005, and for purposes of section 114(1)(*a*) of the Act, you are hereby advised that a child has been abused in a manner causing physical injury/ sexually abused/ deliberately neglected or is in need of care and protection.

Source of report (do not identify person) ☐ **Victim** ☐ **Relative** ☐ **Parent**

☐ **Neighbour** ☐ **friend** ☐ **Professional (specify)** ....

☐ **Other (specify)** ....

**Date Reported to child protection organisation:**	**DD**	**MM**	**CCYY**

1. INFORMANT: (DETAILS OF PERSON WHO REPORTS ALLEGED ABUSE)

**Surname**			**Full name(s)**			
**Gender:**	**M**	**F**	**Date of Birth:**	**DD**	**MM**	**CCYY**
**Age / Estimated Age:**			**Relationship to Child:**			
*** ID no:**			*** Passport no:**			
**Contact no:**						

2. CHILD: (COMPLETE PER CHILD)

**Surname**			**Full name(s)**			
**Gender:**	**M**	**F**	**Date of Birth:**	**DD**	**MM**	**CCYY**
**School Name:**			**Grade:**		**Age / Estimated Age:**	
*** ID no:**			*** Passport no:**			
**Contact no:**						

3. PARENTS OF CHILD (If other than above)

**Surname: Father / Step-father**				**Full name(s)**		
**Date of Birth:**	**DD**	**MM**	**CCYY**	**Gender:**	**M**	**F**
**ID number:**				**Age:**		

Surname: Mother / Step-mother				Full name(s)		
Date of Birth:	DD	MM	CCYY	Gender:	M	F
ID number:				Age:		

Names and ages of siblings or other children if helpful for tracking		
Surname	Full names	Age/Date of birth

Street Address (include postal code):	Postal Code:

4. CAREGIVER INFORMATION (If not same as trusted person or parent(s) of child)	
Surname:	Name:
Physical Address:	Postal address
Relationship to child:	
Telephone number:	Mobile:

5. NATURE OF REPORT

☐ Child abuse ☐ Child labour ☐ Child trafficking ☐Child living and begging on the street

☐ Commercial sexual exploitation ☐ Exploited children☐ Child abduction

6. OTHER INTERVENTION – CONTACT PERSON TRUSTED BY CHILD	
Surname:	Name:
Physical address:	Telephone number:

Other children interviewed: ☐ Yes ☐ No Number:

7. ALLEGED PERPETRATOR						
7.1) Surname				Full Name(s)		
Date of Birth:	DD	MM	CCYY	Gender:	M	F

ID No:	Age:
* Passport No:	* Drivers license number:
Also known as:	Relationship to child:<br>☐Father ☐Mother ☐Grandfather<br>☐Grandmother ☐Step father ☐Step mother<br>☐Foster father ☐Aunt ☐Uncle<br>☐Foster mother ☐ Sibling ☐ Caregiver<br>☐ Professional: social worker/police officer/teacher/caregiver/priest/dr/ volunteer<br>☐ Other (specify)Other (specify)
Street Address (include postal code):<br><br>Postal Code:	

7.2) WHEREABOUTS OF ALLEGED PERPETRATOR:

☐ Section 153 (Request for removal by SAPS) ☐ Still in home

☐ In hospital (Name/Place...)

☐ In detention (Name/Place...)

☐ Living somewhere else (Address...)

☐ Whereabouts unknown ☐ Un-identified

8. ABUSE

Date of Incident:			If date unknown (mark with X here):	Episodic		Reported to CPR:		
DD	MM	CCYY		Yes	No	DD	MM	CCYY

Place of incident: ☐ Child's home ☐ Field ☐ Tavern ☐ School

☐ Friend's place ☐ After school centre ☐ ECD Centre ☐ Neighbour ☐ Private hostel

☐ Child and youth care centre ☐ Foster home ☐ Temporary safe care

☐ temporary respite care ☐ Other (specify)

8.1) TYPE OF ABUSE (Tick only the one that indicates the key motive of intent)

Physical	Emotional	Sexual	Deliberate neglect

8.2) INDICATORS (Check any that apply)

PHYSICAL: ☐ Abrasions ☐ Bruises ☐ Burns/Scalding ☐ Fractures

☐ Other physical illness ☐ Cuts ☐ Welts

☐ Repeated injuries ☐ Fatal injury (date of death)

☐ **Injury to internal organs** ☐ **Head injuries** ☐ **No visible injuries (elaborate)**

☐ **Poisoning (specify)** ☐ **Other Behavioral or physical (specify)**

EMOTIONAL: ☐ **Withdrawal** ☐ **Depression** ☐ **Self destructive aggressive behaviour**

☐ **Corruption through exposure to illegal activities** ☐ **Deprivation of affection**

☐ **Exposure to anti-social activities** ☐ **Exposure to family violence**

☐ **Parent or care giver negative mental condition** ☐ **Inappropriate and continued criticism**

☐ **Humiliation** ☐ **Isolation** ☐ **Threats** ☐ **Development Delays** ☐ **Oppression**

☐ **Rejection** ☐ **Accusations** ☐ **Anxiety** ☐ **Lack of cognitive stimulation**

☐ **Mental, emotional or developmental condition requiring treatment (specify)**

SEXUAL: ☐ **Contact abuse** ☐ **Rape** ☐ **Sodomy**

☐ **Masturbation** ☐ **Oral sex area** ☐ **Molestation**

☐ **Non-contact abuse (flashing, peeping)** ☐ **Irritation, pain, injury to genital**

☐ **Other indicators of sexual molestation or exploitation (specify)**

DELIBERATE NEGLECT: ☐ **Malnutrition** ☐ **Medical** ☐ **Physical** ☐ **Educational**

☐ **Refusal to assume parental responsibility** ☐ **Neglectful supervision** ☐ **Abandonment**

☐ **Child living and begging on the street**

8.3) Indicate overall degree of risk to child:

☐ **Mild** ☐ **Moderate** ☐ **Severe** ☐ **Unknown**

8.4) Where applicable, tick the secondary type of abuse or multiple abuse: ☐ **Yes** ☐ **No**

Sexual	Physical	Emotional	Deliberate Neglect

Brief explanation of occurrence(s) (including a statement describing frequency and duration)

9. MEDICAL INTERVENTION (*)

Examined by:	Treatment received:	Where (name of hospital, clinic, private doctor):	Hospitalised:
☐ Doctor	☐ Yes		☐ For assessment
☐ Reg. Nurse	☐ No		☐ For treatment

			☐ As temporary safe care (place of safety)
Contact person:	Contact person:	Contact person:	Contact person:
Telephone No:	Telephone No:	Telephone No:	Telephone No:

10. CHILDREN'S COURT INTERVENTION (*)			
Removal of child to temporary safe care (Section 152):	Date		
	MM	DD	CCYY
☐ Yes ☐ No			

11. SAPS: (ACTION RELATED TO ALLEGED ABUSER(S)) – (*)				
Reported to SAPS:	Charges laid:	Date		
☐ Yes ☐ No	☐ Yes ☐ No	DD	MM	CCYY

CASE NR	Police Station	Telephone Nr

Name of Police Officer	Rank of Police Officer

12. CHILD KNOWN TO DESIGNATED CHILD PROTECTION ORGANISATION (DCPO)/ SOCIAL DEVELOPMENT (DSD)?		
12.1) Child known to DCPO/DSD: ☐ Yes ☐ No		
Name of DCPO/DSD Office:	Contact number	Reference number

13. DETAILS OF PERSON WHO REPORTS ALLEGED ABUSE (Refers to a professional or mandatory obliged to report child abuse in terms of Section 110(1))		
Surname:	Name:	Name of employer:
CAPACITY		
Employer Address	Work Telephone Nr	Fax Number
Email Address		

(*) = Complete if information is available or applicable

I declare that the particulars set out in the above mentioned statement are true and correct to the best of my knowledge.

Signature of official: ____

Date: ____

**Official Stamp**
